# Using Mobile Phone Data to Assess Socio-Economic Disparities in Unhealthy Food Reliance during the COVID-19 Pandemic

**DOI:** 10.34133/hds.0101

**Published:** 2023-12-15

**Authors:** Charles Alba, Ruopeng An

**Affiliations:** ^1^ Division of Computational & Data Sciences, Washington University in St Louis, St Louis, MO, USA.; ^2^ Brown School, Washington University in St Louis, St Louis, MO, USA.

## Abstract

**Background:** Although COVID-19 has disproportionately affected socio-economically vulnerable populations, research on its impact on socio-economic disparities in unhealthy food reliance remains scarce. **Methods:** This study uses mobile phone data to evaluate the impact of COVID-19 on socio-economic disparities in reliance on convenience stores and fast food. Reliance is defined in terms of the proportion of visits to convenience stores out of the total visits to both convenience and grocery stores, and the proportion of visits to fast food restaurants out of the total visits to both fast food and full-service restaurants. Visits to each type of food outlet at the county level were traced and aggregated using mobile phone data before being analyzed with socio-economic demographics and COVID-19 incidence data. **Results:** Our findings suggest that a new COVID-19 case per 1,000 population decreased a county’s odds of relying on convenience stores by 3.41% and increased its odds of fast food reliance by 0.72%. As a county’s COVID-19 incidence rate rises by an additional case per 1,000 population, the odds of relying on convenience stores increased by 0.01%, 0.02%, and 0.06% for each additional percentage of Hispanics, college-educated residents, and every additional year in median age, respectively. For fast food reliance, as a county’s COVID-19 incidence rate increases by one case per 1,000 population, the odds decreased by 0.003% for every additional percentage of Hispanics but increased by 0.02% for every additional year in the county’s median age. **Conclusion:** These results complement existing literature to promote equitable food environments.

## Introduction

A combination of systemic racism and the lack of investment in healthy food environments for socio-economically disadvantaged populations has resulted in modern-day disparities surrounding the obesity pandemic witnessed throughout the United States [1–3]. Studies suggest that African Americans, Hispanics, Native Americans, lower-income households, and non-college-educated Americans are more susceptible to obesity compared to white, higher-educated, and higher-earning individuals [1–6]. The unprecedented COVID-19 pandemic has further exposed socio-economic inequities in health outcomes across the United States—with studies revealing that African American counties have the highest case–fatality ratio [7], while Hispanic communities experienced the highest COVID-19 infection rates despite being the most restricted in terms of mobility [7,8]. In spite of this amplifying need to examine COVID-19’s impact on socio-economic disparities surrounding unhealthy food reliance, studies concerning this topic remain scarce, potentially due to their reliance on traditional survey methods, which are bounded by limitations. These include non-response biases, such as low response rates, and response biases, like the limited validation of the accuracy in self-reported food consumption surveys [9]. Additionally, substantial administrative costs often confine these surveys to local or state levels [9].

Some existing studies concerning the socio-economic impact of COVID-19 on dietary patterns present mixed findings. For example, while some found that measures like stay-at-home orders [10] led to increased purchases of fresh produce and meats among African Americans [11], who previously reported less healthy diets [4], others indicated widening disparities, such as reduced fresh food purchases by lower-income families [11] and heightened convenience store visits among Native Americans [12].

To overcome these challenges, we utilize mobile phone data to assess the socio-economic disparities associated with unhealthy food reliance during the pandemic. While mobile phone data provide vast insights, concerns mount about the accuracy and fairness of these sources, like SafeGraph in our study. Comparisons with official statistics have sought to address these concerns [13–15]. For example, Sobolev et al. [13] found a strong correlation between protest sizes from SafeGraph’s data and news media estimates. Kupfer et al. [14] confirmed the consistency of SafeGraph’s temporal accuracy in comparing visitation patterns across 6 U.S. national parks. Additionally, Liang et al. [15] compared SafeGraph’s demographic data with official surveys from Yellowstone National Park and found minimal biases across racial, age, and educational-attainment demographics. These findings underscore the reliability of employing mobile phone data to study unhealthy food reliance.

As such, recent efforts have harnessed mobile phone data to track changes in visitation patterns to unhealthy food outlets during the pandemic. For instance, Banerjee et al. [16] observed that visits to fast food surged in rural counties compared to urban counties due to restrictive measures. Ashby [17] noted that although unhealthy eating habits declined during the pandemic, the reduction was less pronounced among populations with higher obesity rates. Perhaps most relevant to our study, Quintero et al. [12] solely analyzed Indian reservations and showed that households opted for convenience stores over grocery stores during the COVID-19 pandemic.

As such, this study leverages SafeGraph’s mobile phone data to comprehensively assess socio-economic disparities in unhealthy food reliance during the COVID-19 pandemic throughout the United States, with a particular focus on the reliance on convenience stores and fast food. We define “convenience store reliance” as the proportion of visits to convenience stores relative to the total visits to both convenience stores and grocery stores. Similarly, “fast food reliance” is the proportion of visits to fast food restaurants relative to the sum of visits to both fast food and full-service restaurants.

We chose this formula based on recent research indicating that convenience stores and fast food often lead to less nutritious food choices when compared to their grocery stores and full-service restaurant counterparts, despite efforts to increase the availability of healthier foods post-2010 [18]. For example, a 2021 study reported that convenience stores achieved healthier food scores that were 70% lower than supermarkets [19]. Moreover, a 2019 study noted an increased risk of coronary artery calcification among residents of neighborhoods with higher concentrations of convenience stores [20]. Similarly, as of 2016, fast food customers achieved a 69.8% “poor” diet quality adherence score, substantially higher than the 52% score for full-service restaurant patrons [21], despite the narrowing disparities.

Our study aims to make the following contributions: Firstly, it seeks to understand socio-economic disparities surrounding fast food and convenience store reliance at a national level across the United States. Most survey-centric literature has been conducted at a local or state level to date. Secondly, it aims to bridge the limited number of studies seeking to understand COVID-19’s impact on socio-economic disparities surrounding fast food and convenience store reliance across the United States. Finally, it promotes the novel use of mobile phone data in addressing socio-economic inequities surrounding built environments and unhealthy food reliance; studies encompassing this topic have been predominantly survey-centric.

## Methods

### Study setting

Our mobile phone data were provided by SafeGraph, a data service provider that aggregates location-based mobile phone data from various third-party service providers. SafeGraph provides census-tract-level data detailing monthly visitors at over 40 million points of interest (POIs) across the United States [22].

These data enable us to assess changes in each county’s monthly visitation patterns to convenience stores, grocery stores, fast food restaurants, and full-service restaurants. Moreover, we can analyze the relationship between these patterns and the corresponding socio-economic demographics across various phases of the COVID-19 pandemic.

In total, our longitudinal study encompasses monthly visitation figures for different types of food stores across 3,141 counties in the United States, covering a span of 41 months from January 2019 to May 2022. This accounts for 99.9% (3,141/3,143) of U.S. counties, excluding Puerto Rico. The 2 counties not included—Chugach and Copper River Census Area—are rural regions in Alaska.

Henceforth, our research aims could be defined as follows:

RQ1. How did COVID-19 impact socio-economic disparities in reliance on convenience stores?

RQ2. How did COVID-19 impact socio-economic disparities in reliance on fast food outlets?

RQ3. What socio-economic disparities influence reliance on convenience stores irrespective of COVID-19?

RQ4. What socio-economic disparities influence reliance on fast food outlets irrespective of COVID-19?

### Experimental design

**Fig. 1.**
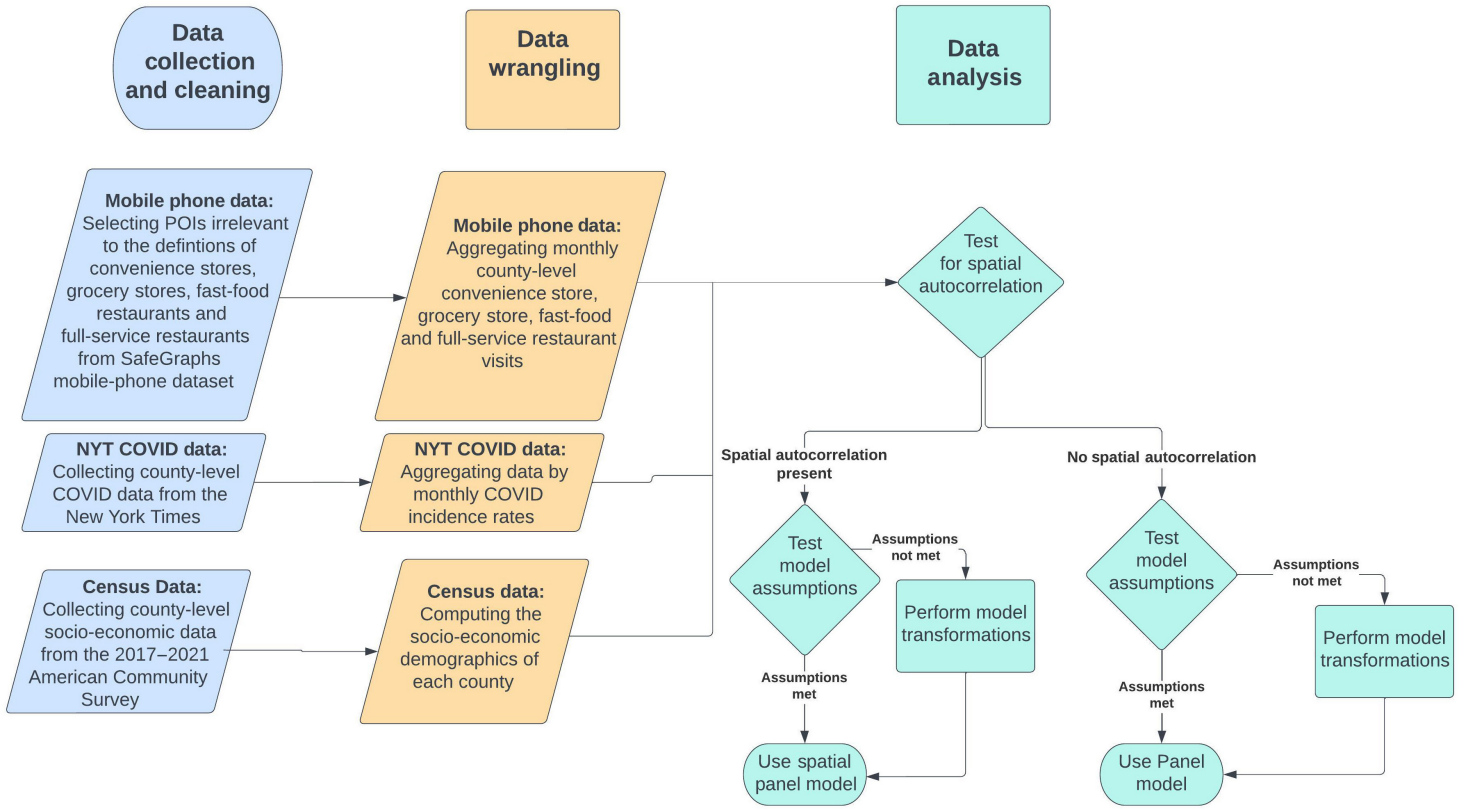
A flowchart illustrating our experimental design.

Figure 1 displays a flowchart illustrating our overall research design.

In summary, we utilized mobile phone data provided by SafeGraph to identify, trace, and aggregate the county origins of visitors to fast food restaurants, full-service restaurants, convenience stores, and grocery stores across the United States. We integrated the mobile phone data with the COVID-19 incidence rates for each county, which were sourced from the *New York Times*, and the socio-economic data obtained from the American Community Survey (ACS), through the 5-digit Federal Information Processing Standard (FIPS) codes. Our analysis employed the logit fixed-effects panel model incorporated with time effects. We selected this model among others through a series of statistical tests, detailed in the “Statistical analysis” section below.

### Variables

#### Dependent variables

Our dependent variables are the county’s monthly level of reliance on convenience stores and fast food restaurants. Each county’s convenience store reliance is calculated as a proportion of monthly-level convenience store visitations over the sum of convenience store and grocery store visitations in the corresponding month within the same county. Meanwhile, each county’s fast food reliance is computed as the proportion of monthly-level fast food visitations over the sum of fast food and full-service restaurant visitations of the corresponding month within the same county. The selection of this formula as a reflection of unhealthy food reliance was detailed in Introduction.

#### Independent variables

Our independent variables included (a) the monthly COVID-19 incidence rate for each county, (b) the socio-economic demographics of each county, and (c) the interaction between each county’s COVID-19 incidence rate and its socio-economic demographics.

**COVID-19 incidence rates**: Each county’s monthly COVID-19 incidence rate was determined by dividing the number of newly reported cases for that month by the county’s total population.

Assessing the exact monthly impact of COVID-19 on individual counties proved challenging due to the diverse implementation of restriction policies at federal, state, and local levels. Nevertheless, it was generally observed that an increase in COVID-19 incidence rates was associated with reduced mobility [10,23,24]. This decline in mobility can be traced back to legislative actions [10] and personal preventive measures [23] adopted during high COVID-19 prevalence. Such legislative actions include directives like social distancing or stay-at-home orders [10]. Conversely, personal preventive measures encompass decisions to stay home or self-quarantine, especially when there is an elevated risk of infection or exposure to COVID-19 [23]. While the magnitude of impact might vary between different COVID-19 waves, the overarching trend was consistent across distinct waves [24].

**Socio-economic demographics**: Socio-economic demographics can be categorized into racial and other socio-economic factors. Racial demographics include the county proportions of non-Hispanic African Americans, Hispanics, non-Hispanic Asian Americans, and non-Hispanic Native Americans. Other socio-economic measures in our study are population density (1,000/km^2^), median household income (in $1,000), median age, and the percentage of residents over 25 with some college education or training. Table S1 lists socio-economic variables that were considered but excluded from our study due to high multi-collinearity, further discussed in the “Statistical analysis” section.

### Materials

#### Mobile phone data

SafeGraph supplied our mobile phone data through their monthly patterns dataset, covering visitor origins to specific POIs across the country, categorized by North American Industry Classification System (NAICS) codes [22]. We received monthly locational data for POIs under NAICS codes 722513 (limited-service restaurants), 722511 (full-service restaurants), 4451 (grocery stores), and 445120 (convenience stores) from January 2019 to May 2022.

To ensure accurate representation of fast food chains, full-service restaurants, grocery stores, and convenience stores, we cross-referenced external sources and curated keywords linked with each store type, as detailed in Table S2.

For instance, SafeGraph sometimes categorizes healthy food franchises, like salad bars, as limited-service restaurants. To focus on restaurants serving high-calorie foods, we adopted methodologies from Athens et al. [25] and An and Sturm [9], using a list from Technomic, a food industry consulting firm [26]. This confirmed the inclusion of chains serving high-calorie items like burgers, tacos, and sugary desserts [9]. Our study used 155,413 POIs from 63 fast food brands. POIs mislabeled as full-service restaurants, but present in Technomic’s fast food list, were excluded, leaving 537,247 POIs from 364,289 full-service restaurant chains.

Additionally, entities like cafes and bakeries were often mislabeled by SafeGraph as grocery or convenience stores. Using a list of keywords in Table S2, we filtered out these mislabeled entities. Our dataset then consisted of 101,676 grocery store POIs across 65,227 brands and 58,121 convenience store POIs across 24,989 brands.

#### Socio-economic data

Our socio-economic data were obtained from the 2017 to 2021 ACS census data [27].

#### COVID-19 incidence data

The COVID-19 incidence data were obtained from the publicly available *New York Times* 2020 to 2022 COVID-19 counties dataset [28]. The incidence rate will be interpreted as the number of new cases in each county in a given month per 1,000 total population of that county.

### Statistical analysis

As mentioned in the “Variables” section, our dependent variables encompass the county’s monthly-level convenience stores and fast food reliance, while our independent variables comprise the county’s monthly-level COVID-19 incidence rate, its socio-economic demographics, and the interaction effects between them. The interaction effects aim to measure the impact of COVID-19 on socio-economic disparities related to fast food and convenience store reliance, per the objectives of RQ1 and RQ2, listed in the “Study setting” section. Concurrently, the socio-economic covariates are employed to analyze socio-economic disparities concerning fast food and convenience stores, regardless of COVID-19’s influence, aligning with the objectives of RQ3 and RQ4.

We analyzed the data using a logit fixed-effects panel model, incorporating time effects, indexed by both county and month. This model choice allows us to control for unobserved heterogeneity, ensuring that temporal variations across months or any inherent characteristics of the counties do not introduce bias into our estimated effects. This model was selected over spatial panel models, the pooled ordinary least square model, and the random-effects panel model based on the results of Moran’s I spatial autocorrelation test, the *F*-test, and the Hausman Chi-square test, respectively. The results of these tests can be referenced in Table S3.

Statistical evaluations, conducted to verify our model’s assumptions, are showcased in Table S4. In particular, the logit transformation was applied to meet the assumptions of normality. Furthermore, robust standard errors were employed to address potential concerns surrounding homoscedasticity and cross-sectionality. The independent variables were selected to ensure that multi-collinearity, measured by the variance inflation factor (VIF) scores, was held within acceptable thresholds. Variables that were considered, but are omitted due to multi-collinearity, are listed in Table S1.

## Results

**Fig. 2.**
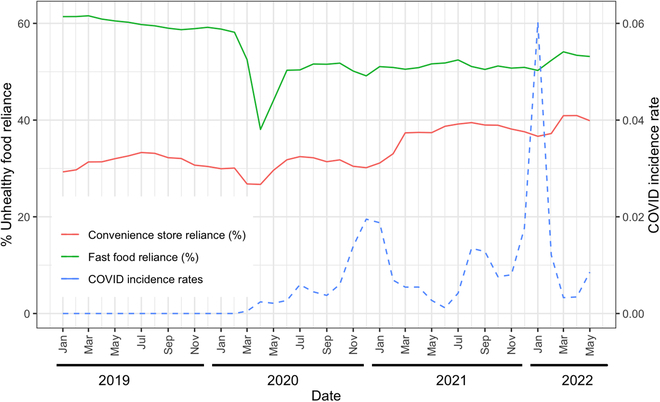
Change in convenience store reliance, fast food reliance, and the COVID-19 incidence rate throughout the United States from January 2019 to May 2022.

Tables 1 through 4 provide summary statistics regarding the impact of COVID-19 on the reliance on convenience stores and fast food outlets. Additionally, these tables illustrate visitation rates to convenience stores, grocery stores, fast food restaurants, and full-service restaurants, stratified by the predominant socio-economic category of each county in the United States. These statistics show the mean values and confidence intervals derived from longitudinal data encompassing all 3,141 US counties over 41 months, from January 2019 to May 2022. The categorization of low and high COVID-19 incidence rates is determined based on thresholds set at the 10th and 90th percentiles of COVID-19 incidence rates throughout these 41 months across all counties. These summary statistics are complemented by Fig. 2, which displays the overarching trends in convenience stores and fast food reliance from January 2019 to May 2022.

**Table 1.** Summary statistics of convenience store and fast food reliance by the predominant race of counties in the United States. The 10th and 90th percentile of COVID-19 incidence rates were calculated based on county-month values (i.e., 3,141 counties × 41 months).

Predominant race category in a county^a^	Number of counties	Store type	Statistics	Mean (95% confidence interval)		
				Low COVID-19 incidence rate^b^	High COVID-19 incidence rate^c^	Overall
White	2,843	Convenience store	Convenience store reliance^d^	0.39 (0.39, 0.39)	0.41 (0.4, 0.41)	0.4 (0.4, 0.4)
African American	130			0.37 (0.35, 0.38)	0.38 (0.36, 0.39)	0.37 (0.37, 0.38)
Hispanic	128			0.29 (0.28, 0.3)	0.37 (0.36, 0.38)	0.35 (0.34, 0.35)
Asian American	5			0.08 (0.04, 0.12)	0.15 (0.09, 0.2)	0.18 (0.17, 0.19)
Native American	35			0.1 (0.07, 0.13)	0.3 (0.27, 0.33)	0.31 (0.3, 0.32)
Overall	3,141			0.38 (0.38, 0.38)	0.4 (0.4, 0.41)	0.39 (0.39, 0.39)
White	2,843	Convenience store	Convenience store visits per 1,000 population	34.92 (34.41, 35.43)	40.07 (39.36, 40.79)	36.61 (36.39, 36.83)
African American	130			23.19 (22.29, 24.1)	27.36 (26.11, 28.61)	26.64 (26.24, 27.04)
Hispanic	128			16.82 (15.74, 17.91)	34.43 (32.2, 36.66)	30.6 (29.91, 31.28)
Asian American	5			3.12 (1.35, 4.89)	5.51 (3.25, 7.78)	6.92 (6.35, 7.5)
Native American	35			0.98 (0.71, 1.26)	7.82 (5.77, 9.87)	8.22 (7.63, 8.82)
Overall	3,141			32.56 (32.09, 33.03)	38.85 (38.19, 39.51)	35.59 (35.38, 35.79)
White	2,843	Convenience store	Grocery store visits per 1,000 population	45.05 (44.72, 45.39)	49.14 (48.56, 49.72)	46.55 (46.39, 46.7)
African American	130			41.83 (40.49, 43.18)	44.09 (42.74, 45.45)	43.74 (43.31, 44.17)
Hispanic	128			39.51 (38.28, 40.74)	53.48 (51.12, 55.84)	52.43 (51.62, 53.24)
Asian American	5			20.05 (15.21, 24.88)	21.5 (15.02, 27.98)	27.11 (25.49, 28.74)
Native American	35			7.44 (6.47, 8.41)	11.69 (10.07, 13.3)	13.13 (12.62, 13.64)
Overall	3,141			43.98 (43.66, 44.31)	48.55 (48.01, 49.09)	46.27 (46.12, 46.41)
White	2,843	Fast food	Fast food reliance^e^	0.57 (0.57, 0.57)	0.49 (0.49, 0.49)	0.52 (0.52, 0.52)
African American	130			0.52 (0.52, 0.53)	0.44 (0.44, 0.45)	0.47 (0.47, 0.47)
Hispanic	128			0.59 (0.59, 0.6)	0.49 (0.48, 0.49)	0.53 (0.52, 0.53)
Asian American	5			0.86 (0.82, 0.9)	0.66 (0.62, 0.71)	0.7 (0.68, 0.71)
Native American	35			0.76 (0.73, 0.8)	0.54 (0.52, 0.56)	0.57 (0.56, 0.57)
Overall	3,141			0.57 (0.57, 0.57)	0.49 (0.49, 0.49)	0.52 (0.52, 0.52)
White	2,843	Fast food	Fast food visits per 1,000 population	182.71 (181.24, 184.18)	158.98 (156.91, 161.05)	162.62 (161.96, 163.27)
African American	130			160.39 (155.62, 165.16)	129.98 (126.11, 133.85)	136.32 (134.91, 137.73)
Hispanic	128			142.24 (137.62, 146.86)	156.28 (149.09, 163.48)	172.89 (170.17, 175.6)
Asian American	5			75.3 (42.09, 108.5)	68.32 (44.13, 92.51)	107.14 (98.14, 116.15)
Native American	35			23.27 (19.05, 27.49)	35.3 (29.16, 41.44)	41.91 (40.02, 43.8)
Overall	3,141			177.59 (176.18, 179)	155.92 (153.99, 157.84)	160.51 (159.9, 161.13)
White	2,843	Fast food	Full-service restaurant visits per 1,000 population	148.64 (147.19, 150.08)	171.28 (169.27, 173.29)	155.92 (155.32, 156.52)
African American	130			144.13 (140.24, 148.03)	163.61 (158.83, 168.4)	152.16 (150.58, 153.74)
Hispanic	128			96 (93.05, 98.95)	160.11 (153.2, 167.01)	153.39 (151.1, 155.67)
Asian American	5			23.58 (10.41, 36.75)	43.71 (27.11, 60.31)	50.16 (46.16, 54.16)
Native American	35			8.03 (6.33, 9.72)	35.8 (29.16, 42.44)	37.55 (35.52, 39.57)
Overall	3,141			144.7 (143.33, 146.06)	168.37 (166.49, 170.25)	154.18 (153.62, 154.74)

^a^ The predominant race in a county is the largest racial group in a county.

^b^ The 10th percentile of COVID-19 incidence rates was calculated across county-month values, meaning it was computed across all counties over all months, encompassing all 41 months across all 3,141 counties (i.e., 3,141 counties × 41 months).

^c^ The 90th percentile of COVID-19 incidence rates was calculated across county-month values, meaning it was computed across all counties over all months, encompassing all 41 months across all 3,141 counties (i.e., 3,141 counties × 41 months).

^d^ The proportion of convenience store visits over the sum of both convenience store and grocery store visits among residents of a county.

^e^ The proportion of fast food visits over the sum of both fast food and full-service restaurant visits among residents of a county.

**Table 2.** Summary statistics of convenience store and fast food reliance by the predominant income of counties in the United States. The 10th and 90th percentile of COVID-19 incidence rates were calculated based on county-month values (i.e., 3,141 counties × 41 months).

Predominant income category in a county^a^	Number of counties	Store type	Statistics	Mean (95% confidence interval)		
				Low COVID-19 incidence rate^b^	High COVID-19 incidence rate^c^	Overall
<$30,000	523	Convenience store	Convenience store reliance^d^	0.4 (0.39, 0.4)	0.39 (0.39, 0.4)	0.38 (0.38, 0.39)
Between $30,000 and $59,999	1,022			0.42 (0.41, 0.42)	0.42 (0.42, 0.43)	0.41 (0.41, 0.42)
Between $60,000 and $99,999	394			0.38 (0.37, 0.39)	0.45 (0.44, 0.45)	0.43 (0.43, 0.43)
≥$100,000	1,202			0.27 (0.26, 0.27)	0.35 (0.34, 0.35)	0.34 (0.33, 0.34)
Overall	3,141			0.38 (0.38, 0.38)	0.4 (0.4, 0.41)	0.39 (0.39, 0.39)
<$30,000	523	Convenience store	Convenience store visits per 1,000 population	31.94 (31.26, 32.61)	37.67 (36.85, 38.49)	34.09 (33.84, 34.34)
Between $30,000 and $59,999	1,022			37.5 (36.64, 38.35)	41.81 (40.27, 43.35)	38.61 (38.14, 39.08)
Between $60,000 and $99,999	394			36.95 (35.31, 38.59)	44.97 (43.28, 46.66)	41.98 (41.42, 42.53)
≥$100,000	1,202			20.81 (19.92, 21.7)	29.78 (28.72, 30.85)	28.31 (27.98, 28.64)
Overall	3,141			32.56 (32.09, 33.03)	38.85 (38.19, 39.51)	35.59 (35.38, 35.79)
<$30,000	523	Convenience store	Grocery store visits per 1,000 population	43.35 (42.83, 43.88)	50.75 (50.13, 51.37)	47.49 (47.29, 47.68)
Between $30,000 and $59,999	1,022			42.86 (42.33, 43.38)	47.35 (45.99, 48.71)	44.93 (44.59, 45.27)
Between $60,000 and $99,999	394			45.74 (44.76, 46.73)	46.83 (45.79, 47.87)	45.45 (45.11, 45.79)
≥$100,000	1,202			46.16 (45.32, 47)	46.57 (45.65, 47.48)	46.69 (46.41, 46.97)
Overall	3,141			43.98 (43.66, 44.31)	48.55 (48.01, 49.09)	46.27 (46.12, 46.41)
<$30,000	523	Fast food	Fast food reliance^e^	0.54 (0.54, 0.54)	0.47 (0.47, 0.47)	0.5 (0.5, 0.5)
Between $30,000 and $59,999	1,022			0.57 (0.57, 0.58)	0.49 (0.49, 0.49)	0.52 (0.52, 0.52)
Between $60,000 and $99,999	394			0.58 (0.57, 0.59)	0.5 (0.5, 0.51)	0.53 (0.53, 0.53)
≥$100,000	1,202			0.62 (0.62, 0.63)	0.52 (0.52, 0.52)	0.55 (0.55, 0.55)
Overall	3,141			0.57 (0.57, 0.57)	0.49 (0.49, 0.49)	0.52 (0.52, 0.52)
<$30,000	523	Fast food	Fast food visits per 1,000 population	180.99 (178.78, 183.21)	164.66 (162.54, 166.78)	165.56 (164.82, 166.31)
Between $30,000 and $59,999	1,022			166.29 (164.05, 168.53)	150.92 (146.04, 155.79)	153.83 (152.37, 155.29)
Between $60,000 and $99,999	394			173.11 (168.95, 177.27)	145.3 (141.86, 148.74)	152.67 (151.43, 153.9)
≥$100,000	1,202			196.54 (192.6, 200.49)	152.38 (148.84, 155.93)	167.89 (166.64, 169.13)
Overall	3,141			177.59 (176.18, 179)	155.92 (153.99, 157.84)	160.51 (159.9, 161.13)
<$30,000	523	Fast food	Full-service restaurant visits per 1,000 population	162 (159.87, 164.12)	188.74 (186.34, 191.14)	171.78 (171, 172.57)
Between $30,000 and $59,999	1,022			131.78 (129.73, 133.83)	159.08 (154.79, 163.36)	144.97 (143.78, 146.16)
Between $60,000 and $99,999	394			138.07 (133.91, 142.24)	149.46 (145.49, 153.42)	140 (138.7, 141.29)
≥$100,000	1,202			135.23 (131.27, 139.18)	149.83 (145.91, 153.75)	142.38 (141.15, 143.61)
Overall	3,141			144.7 (143.33, 146.06)	168.37 (166.49, 170.25)	154.18 (153.62, 154.74)

^a^ The predominant income group in a county represents the largest population segment within a specific household income category.

^b^ The 10th percentile of COVID-19 incidence rates was calculated across county-month values, meaning it was computed across all counties over all months, encompassing all 41 months across all 3,141 counties (i.e., 3,141 counties × 41 months).

^c^ The 90th percentile of COVID-19 incidence rates was calculated across county-month values, meaning it was computed across all counties over all months, encompassing all 41 months across all 3,141 counties (i.e., 3,141 counties × 41 months).

^d^ The proportion of convenience store visits over the sum of both convenience store and grocery store visits among residents of a county.

^e^ The proportion of fast food visits over the sum of both fast food and full-service restaurant visits among residents of a county.

**Table 3.** Summary statistics of convenience store and fast food reliance by the highest education category of counties in the United States. The 10th and 90th percentile of COVID-19 incidence rates were calculated based on county-month values (i.e., 3,141 counties × 41 months).

Predominant highest educational attainment category in a county^a^	Number of counties	Store type	Statistics	Mean (95% confidence interval)		
				Low COVID-19 incidence rate^b^	High COVID-19 incidence rate^c^	Overall
Less than high school diploma	3	Convenience store	Convenience store reliance^d^	0.36 (0.25, 0.46)	0.34 (0.27, 0.4)	0.28 (0.24, 0.31)
High school diploma or equivalent	3,119			0.4 (0.4, 0.41)	0.38 (0.38, 0.38)	0.39 (0.39, 0.39)
Bachelor’s degree	10			0.21 (0.19, 0.24)	0.18 (0.17, 0.19)	0.2 (0.19, 0.2)
Advanced degree	9			0.27 (0.25, 0.3)	0.32 (0.31, 0.34)	0.28 (0.27, 0.29)
Overall	3,141			0.4 (0.4, 0.41)	0.38 (0.38, 0.38)	0.39 (0.39, 0.39)
Less than high school diploma	3	Convenience store	Convenience store visits per 1,000 population	56.98 (21.49, 92.47)	14.31 (7.31, 21.31)	31.16 (22.83, 39.49)
High school diploma or equivalent	3,119			38.95 (38.28, 39.61)	32.74 (32.27, 33.21)	35.75 (35.54, 35.95)
Bachelor’s degree	10			10.11 (7.65, 12.57)	7.06 (6.56, 7.56)	9.28 (8.5, 10.06)
Advanced degree	9			8.24 (7.18, 9.3)	20.19 (18.47, 21.92)	11.02 (10.41, 11.62)
Overall	3,141			38.85 (38.19, 39.51)	32.56 (32.09, 33.03)	35.59 (35.38, 35.79)
Less than high school diploma	3	Convenience store	Grocery store visits per 1,000 population	88.47 (46.9, 130.04)	37.53 (17.7, 57.37)	69.41 (56.64, 82.18)
High school diploma or equivalent	3,119			48.59 (48.05, 49.13)	44.04 (43.72, 44.37)	46.34 (46.19, 46.49)
Bachelor’s degree	10			33.31 (27.58, 39.04)	31.25 (30.33, 32.17)	32.73 (30.99, 34.47)
Advanced degree	9			22.49 (19.87, 25.12)	41.83 (39.57, 44.08)	28.52 (27.34, 29.71)
Overall	3,141			48.55 (48.01, 49.09)	43.98 (43.66, 44.31)	46.27 (46.12, 46.41)
Less than high school diploma	3	Fast food	Fast food reliance^e^	0.51 (0.46, 0.55)	0.62 (0.6, 0.65)	0.55 (0.53, 0.57)
High school diploma or equivalent	3,119			0.49 (0.49, 0.49)	0.57 (0.57, 0.57)	0.52 (0.52, 0.52)
Bachelor’s degree	10			0.64 (0.6, 0.68)	0.78 (0.76, 0.8)	0.68 (0.67, 0.69)
Advanced degree	9			0.58 (0.56, 0.59)	0.67 (0.66, 0.68)	0.6 (0.6, 0.61)
Overall	3,141			0.49 (0.49, 0.49)	0.57 (0.57, 0.57)	0.52 (0.52, 0.52)
Less than high school diploma	3	Fast food	Fast food visits per 1,000 population	268.34 143.19, 393.5)	152.44 (87.36, 217.52)	211.39 (172.18, 250.59)
High school diploma or equivalent	3,119			155.95 (154.02, 157.88)	177.44 (176.03, 178.86)	160.66 (160.05, 161.28)
Bachelor’s degree	10			121.89 (101.46, 142.32)	178.85 (162.38, 195.31)	144.51 (136.06, 152.95)
Advanced degree	9			72.88 (66.07, 79.68)	193.33 (181.49, 205.16)	109.21 (103.23, 115.19)
Overall	3,141			155.92 (153.99, 157.84)	177.59 (176.18, 179)	160.51 (159.9, 161.13)
Less than high school diploma	3	Fast food	Full-service restaurant visits per 1,000 population	263.63 (137.4, 389.86)	105.39 (45.44, 165.34)	178.87 (144.3, 213.44)
High school diploma or equivalent	3,119			168.59 (166.7, 170.47)	145.2 (143.83, 146.57)	154.63 (154.07, 155.2)
Bachelor’s degree	10			91.74 (65.85, 117.63)	47.8 (43.9, 51.7)	82.87 (74.88, 90.87)
Advanced degree	9			54.94 (47.51, 62.37)	94.18 (88.86, 99.49)	66.69 (63.98, 69.39)
Overall	3,141			168.37 (166.49, 170.25)	144.7 (143.33, 146.06)	154.18 (153.62, 154.74)

^a^ The predominant educational attainment in a county represents the level of education achieved by the majority of its population.

^b^ The 10th percentile of COVID-19 incidence rates was calculated across county-month values, meaning it was computed across all counties over all months, encompassing all 41 months across all 3,141 counties (i.e., 3,141 counties × 41 months).

^c^ The 90th percentile of COVID-19 incidence rates was calculated across county-month values, meaning it was computed across all counties over all months, encompassing all 41 months across all 3,141 counties (i.e., 3,141 counties × 41 months).

^d^ The proportion of convenience store visits over the sum of both convenience store and grocery store visits among residents of a county.

^e^ The proportion of fast food visits over the sum of both fast food and full-service restaurant visits among residents of a county.

**Table 4.** Summary statistics of convenience store and fast food reliance by the predominant age category and population density of counties in the United States. The 10th and 90th percentile of COVID-19 incidence rates were calculated based on county-month values (i.e., 3,141 counties × 41 months).

Predominant SES category in a county^a^^,^^b^	Number of counties	Store type	Statistics	Mean (95% confidence interval)		
				Low COVID-19 incidence rate^c^	High COVID-19 incidence rate^d^	Overall
Low population density	2,799	Convenience store	Convenience store reliance^e^	0.39 (0.39, 0.4)	0.41 (0.41, 0.42)	0.4 (0.4, 0.4)
Medium population density	209			0.29 (0.28, 0.29)	0.31 (0.3, 0.32)	0.31 (0.31, 0.32)
High population density	133			0.25 (0.24, 0.26)	0.29 (0.28, 0.3)	0.3 (0.29, 0.3)
Age less than 55	2,028			0.36 (0.36, 0.36)	0.39 (0.39, 0.4)	0.39 (0.39, 0.39)
Age more than 55	1,113			0.41 (0.41, 0.42)	0.42 (0.42, 0.43)	0.4 (0.4, 0.4)
Overall	3,141			0.38 (0.38, 0.38)	0.4 (0.4, 0.41)	0.39 (0.39, 0.39)
Low population density	2,799	Convenience store	Convenience store visits per 1,000 population	33.86 (33.35, 34.37)	40.44 (39.72, 41.16)	37.12 (36.9, 37.35)
Medium population density	209			22.89 (21.97, 23.8)	25.17 (24.04, 26.3)	25.36 (24.99, 25.73)
High population density	133			18.72 (17.42, 20.03)	17.72 (16.59, 18.84)	19.34 (18.95, 19.73)
Age less than 55	2,028			29.67 (29.15, 30.19)	38.02 (37.39, 38.64)	35.42 (35.22, 35.61)
Age more than 55	1,113			36.63 (35.76, 37.49)	40.45 (38.99, 41.92)	35.9 (35.46, 36.34)
Overall	3,141			32.56 (32.09, 33.03)	38.85 (38.19, 39.51)	35.59 (35.38, 35.79)
Low population density	2,799	Convenience store	Grocery store visits per 1,000 population	43.13 (42.78, 43.48)	48.86 (48.26, 49.46)	46.28 (46.11, 46.44)
Medium population density	209			52.88 (51.99, 53.78)	50.16 (49.11, 51.22)	49.85 (49.53, 50.18)
High population density	133			48.43 (47.56, 49.3)	37.93 (37.07, 38.78)	40.42 (40.1, 40.74)
Age less than 55	2,028			44.57 (44.18, 44.97)	49.8 (49.34, 50.25)	47.81 (47.67, 47.95)
Age more than 55	1,113			42.37 (41.81, 42.93)	46.06 (44.78, 47.34)	43.45 (43.13, 43.77)
Overall	3,141			43.98 (43.66, 44.31)	48.55 (48.01, 49.09)	46.27 (46.12, 46.41)
Low population density	2,799	Fast food	Fast food reliance^f^	0.57 (0.56, 0.57)	0.49 (0.49, 0.49)	0.52 (0.52, 0.52)
Medium population density	209			0.57 (0.57, 0.57)	0.49 (0.49, 0.49)	0.53 (0.52, 0.53)
High population density	133			0.64 (0.63, 0.64)	0.53 (0.53, 0.54)	0.57 (0.57, 0.58)
Age less than 55	2,028			0.56 (0.56, 0.56)	0.48 (0.48, 0.48)	0.51 (0.51, 0.51)
Age more than 55	1,113			0.58 (0.58, 0.59)	0.5 (0.5, 0.51)	0.53 (0.53, 0.54)
Overall	3,141			0.57 (0.57, 0.57)	0.49 (0.49, 0.49)	0.52 (0.52, 0.52)
Low population density	2,799	Fast food	Fast food visits per 1,000 population	172.29 (170.78, 173.79)	157.23 (155.11, 159.35)	160.19 (159.52, 160.86)
Medium population density	209			229.77 (225.11, 234.42)	157.34 (152.91, 161.77)	174.23 (172.63, 175.82)
High population density	133			212.22 (208.29, 216.15)	117.42 (113.82, 121.02)	145.82 (144.16, 147.49)
Age less than 55	2,028			189.18 (187.37, 190.98)	165.56 (163.9, 167.22)	173.12 (172.54, 173.7)
Age more than 55	1,113			154.56 (152.46, 156.65)	136.43 (131.96, 140.89)	137.55 (136.21, 138.88)
Overall	3,141			177.59 (176.18, 179)	155.92 (153.99, 157.84)	160.51 (159.9, 161.13)
Low population density	2,799	Fast food	Full-service restaurant visits per 1,000 population	142.83 (141.37, 144.3)	170.69 (168.65, 172.74)	155.97 (155.36, 156.58)
Medium population density	209			179.82 (175.01, 184.64)	167.56 (162.5, 172.63)	159.54 (157.92, 161.15)
High population density	133			123.39 (119.64, 127.13)	108.27 (104.07, 112.46)	108.05 (106.72, 109.38)
Age less than 55	2,028			159.84 (158.08, 161.61)	183.3 (181.42, 185.17)	170.54 (169.94, 171.15)
Age more than 55	1,113			117.48 (115.59, 119.37)	138.69 (134.76, 142.61)	124.35 (123.28, 125.43)
Overall	3,141			144.7 (143.33, 146.06)	168.37 (166.49, 170.25)	154.18 (153.62, 154.74)

^a^ The predominant age group in a county represents the largest age category within that county.

^b^ Low population density counties are counties with less than 1,000 population/km^2^, medium population density counties are counties with between 1,000 and 3,000 population/km^2^, while high population density counties are counties with more than 3,000 population/km^2^.

^c^ The 10th percentile of COVID-19 incidence rates was calculated across county-month values, meaning it was computed across all counties over all months, encompassing all 41 months across all 3,141 counties (i.e., 3,141 counties × 41 months).

^d^ The 90th percentile of COVID-19 incidence rates was calculated across county-month values, meaning it was computed across all counties over all months, encompassing all 41 months across all 3,141 counties (i.e., 3,141 counties × 41 months).

^e^ The proportion of convenience store visits over the sum of both convenience store and grocery store visits among residents of a county.

^f^ The proportion of fast food visits over the sum of both fast food and full-service restaurant visits among residents of a county.

**Fig. 3.**
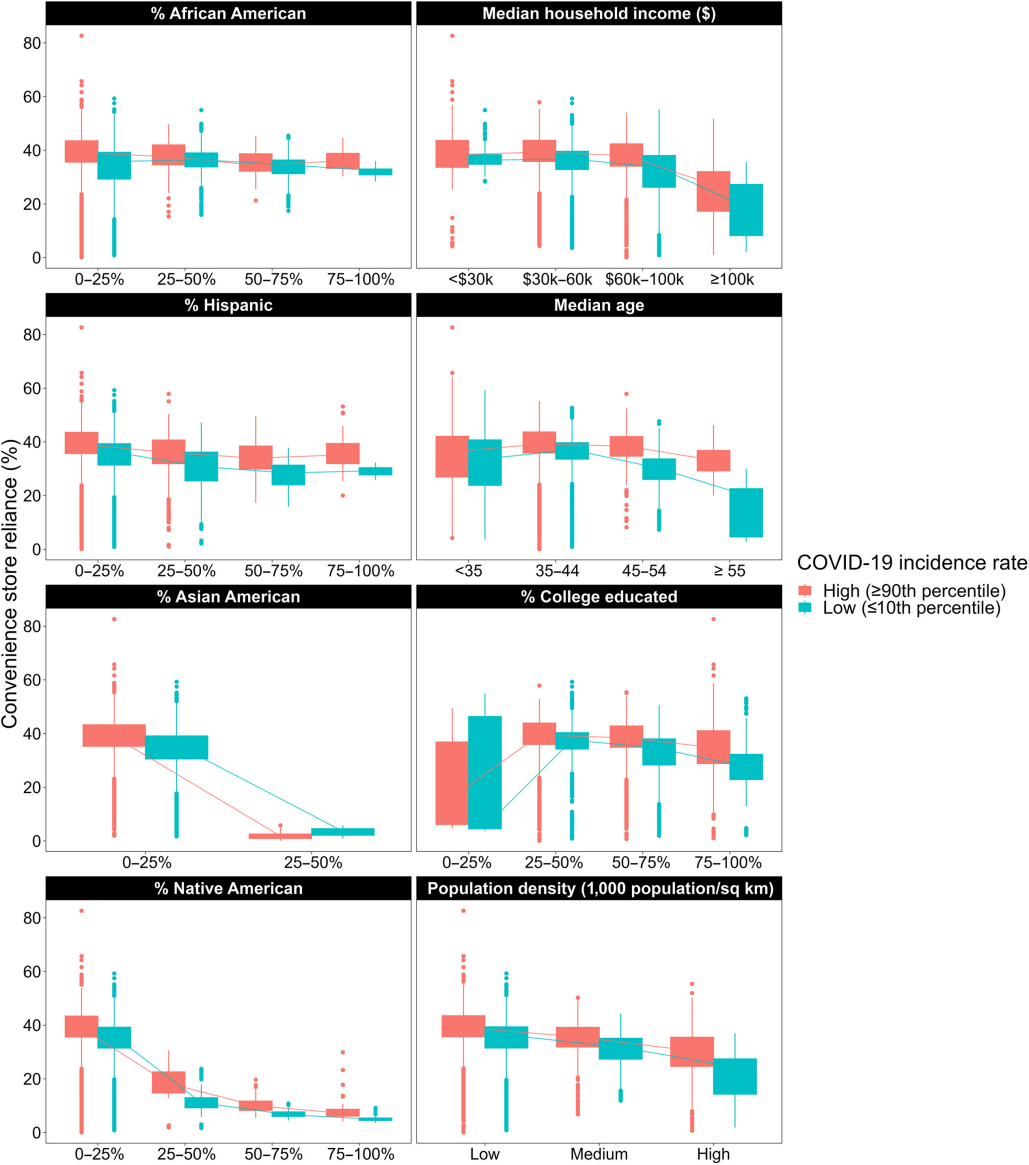
Boxplots of convenience store reliance by county-level socio-economic demographic under low vs. high COVID-19 incidence rates.

**Fig. 4.**
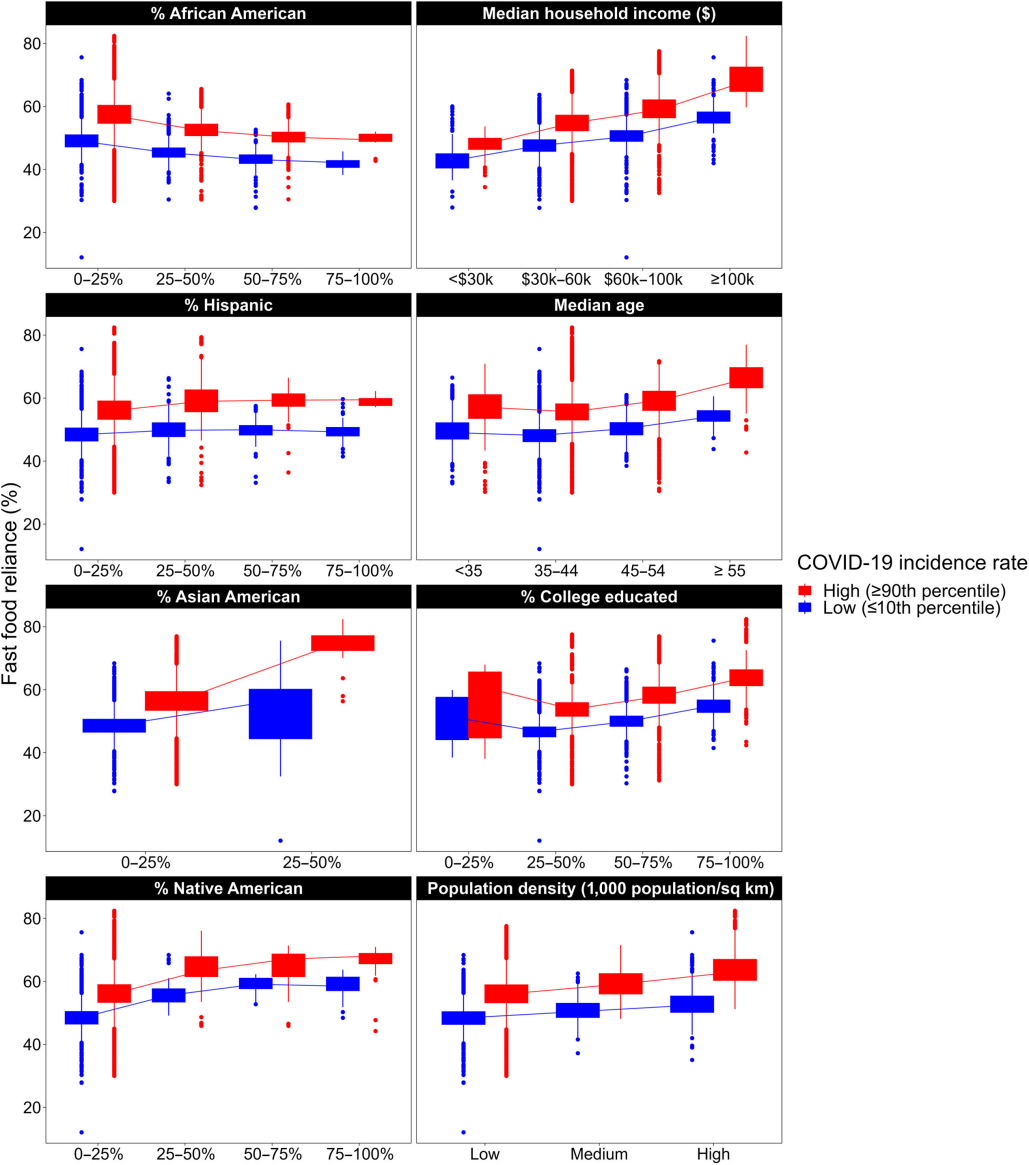
Boxplots of fast food reliance by county-level socio-economic demographic under low vs. high COVID-19 incidence rates.

On the other hand, Table 5 showcases the outcomes of our statistical analysis. Figures 3 and 4 offer a visual representation of the results, emphasizing the impact of COVID-19 on convenience stores and fast food reliance.

**Table 5.** Estimated convenience store/fast food reliance relative to monthly county-level COVID-19 incidence rate and socio-economic distributions. Racial and college-educated demographics are presented in the form of a proportion. COVID-19 incidence rates are the rate of new COVID-19 cases per population per county. ***P < 0.001, **P < 0.01, *P < 0.05.

Independent variable	Convenience store reliance^a^	Fast food reliance^b^
	Coefficient (robust standard error)	Coefficient (robust standard error)
COVID-19 incidence rate	−34.719 ***	7.191 **
	(7.838)	(2.413)
African American	−0.523 ***	−0.188 ***
	(0.109)	(0.030)
Hispanic	−0.900 ***	0.367 ***
	(0.120)	(0.035)
Native American	−3.775 ***	1.033 ***
	(0.736)	(0.183)
Asian American	−8.129 ***	1.737 **
	(1.848)	(0.598)
Population density (1,000 population/km^2^)	0.000	0.018 ***
	(0.003)	(0.004)
Median household income (per $1,000)	−0.001	0.007 ***
	(0.002)	(0.002)
Median age	−0.039 ***	0.017 ***
	(0.005)	(0.002)
College educated	−0.561 *	0.597 ***
	(0.254)	(0.074)
COVID-19 × African American	−0.948	0.612
	(1.952)	(0.562)
COVID-19 × Hispanic	13.205 ***	−2.597 ***
	(2.522)	(0.669)
COVID-19 × Asian American	−49.21	−54.159
	(50.919)	(42.150)
COVID-19 × Native American	20.53	−7.107
	(10.884)	(5.286)
COVID-19 × population density	0.261	0.208
	(0.368)	(0.186)
COVID-19 × median household income	−0.043	0.022
	(0.046)	(0.016)
COVID-19 × median age	0.602 ***	0.165 *
	(0.175)	(0.080)
COVID-19 × college educated	23.151 ***	1.899
	(5.649)	(2.273)

^a^ Convenience store reliance is the logit proportion of convenience store visits over the sum of both convenience store and grocery store visits among residents of a county.

^b^ Fast food reliance is the logit proportion of convenience store visits over the sum of both convenience store and grocery store visits among residents of a county.

### RQ1: How did COVID-19 impact socio-economic disparities in reliance on convenience stores?

In terms of summary statistics, higher convenience store reliance of 0.4 (CI: [0.4, 0.41]) was witnessed during periods of high COVID-19 incidence rates compared to 0.38 (CI: [0.38, 0.38]) during periods of low incidence as detailed in Table 1.

The results from our statistical analysis, presented in Table 5, indicate that COVID-19 resulted in a significant decrease in convenience store reliance, with every additional COVID-19 case per 1,000 population decreasing the county’s odds of convenience store reliance by 3.41%. A detailed version of our statistical analysis, including odds calculations, can be found in the Supplementary Materials.

#### Racial disparities

In terms of summary statistics, Table 1 indicates that convenience store reliance was higher during periods of high COVID-19 incidence across racial groups. In predominantly white counties, reliance was higher at 0.41 (CI: [0.40, 0.41]) during high incidence periods compared to 0.39 (CI: [0.39, 0.39]) during low incidence periods. Similarly, for African American counties, reliance was 0.38 (CI: [0.36, 0.39]) compared to 0.37 (CI: [0.35, 0.38]); in Hispanic counties, it was 0.37 (CI: [0.36, 0.38]) compared to 0.29 (CI: [0.28, 0.30]); in Asian American counties, it was 0.15 (CI: [0.09, 0.20]) compared to 0.08 (CI: [0.04, 0.12]); and in Native American counties, it was 0.30 (CI: [0.27, 0.33]) compared to 0.10 (CI: [0.07, 0.13]).

Statistical analysis from Table 5 indicates that increases in COVID-19 incidence rates correspond with significant increases in convenience store reliance among counties with higher Hispanic populations. To provide context to our findings: if a county’s COVID-19 incidence rate goes up by 1 new case per 1,000 population, each additional percentage point in the Hispanic population increased the odds of convenience store reliance relatively by 0.01%. This relationship is illustrated in Fig. 3, which underscores the widening disparities in convenience store reliance as the Hispanic population percentage grows during periods of high COVID-19 incidence rates.

#### Other socio-economic disparities

Summary statistics from Table 2 illustrate the differences in convenience store reliance across counties with distinct predominant household income level groups. In counties with predominantly lower household incomes (income < $30,000), the reliance was 0.39 (CI: [0.39, 0.4]) during periods of high COVID-19 incidence rates, which was lower compared to 0.4 (CI: [0.39, 0.4]) during periods of low incidence rates. For counties with predominantly medium household incomes ($30, 000 ≤ income < $60, 000), reliance was consistently at 0.42. The 2 categories of higher household income counties, those with median incomes from $60,000 to $100,000, and those with median incomes >$100,000 showed higher reliance during periods of high COVID-19 incidence rates at 0.45 (CI: [0.44, 0.45]), compared to 0.38 (CI: [0.37, 0.39]), and 0.35 (CI: [0.34, 0.35]), compared to 0.27 (CI: [0.26, 0.27]), respectively.

As shown in Table 3, counties where the majority of the population holds less than a high school diploma, a high school diploma, or a bachelor’s degree showed lower convenience store reliance during periods of high COVID-19 incidence rates. Specifically, reliance was 0.34 (CI: [0.27, 0.4]) during periods of high COVID-19 incidence compared to 0.36 (CI: [0.25, 0.46]) during periods of low incidence for less than high school. For counties where majority holds high school diplomas, it was 0.38 (CI: [0.38, 0.38]) compared to 0.4 (CI: [0.4, 0.41]); for counties where majority are bachelor degree holders, it was 0.18 (CI: [0.17, 0.19]) compared to 0.21 (CI: [0.19, 0.2]). However, in counties where the majority holds advanced degrees, reliance was higher at 0.32 (CI: [0.31, 0.34]) during high incidence rates, compared to 0.27 (CI: [0.25, 0.3]).

Per Table 4, convenience store reliance was higher during periods of high COVID-19 incidence across all population density categories and age brackets. For low-density counties, reliance was 0.41 (CI: [0.41, 0.42]) during high COVID-19 incidence rates compared to 0.39 (CI: [0.39, 0.4]) during low incidence periods. In medium-density counties, it was 0.31 (CI: [0.3, 0.32]) compared to 0.29 (CI: [0.28, 0.29]); and in high-density counties, it was 0.29 (CI: [0.28, 0.3]) compared to 0.25 (CI: [0.24, 0.26]). For younger counties (majority under 55), reliance was 0.39 (CI: [0.39, 0.4]) during high incidence, compared to 0.36 (CI: [0.36, 0.36]). Similarly, in older counties (majority over 55), reliance was 0.42 (CI: [0.42, 0.43]) compared to 0.41 (CI: [0.41, 0.42]).

Our statistical analysis, as presented in Table 5, demonstrates that an increase in COVID-19 incidence rates led to significant increases in convenience store reliance in counties with older median ages and higher proportions of college-educated residents. Specifically, for every COVID-19 case per 1,000 population, the odds of convenience store reliance increased relatively by 0.02% for every additional percentage point in the college-educated population, and 0.06% for each additional year in median age. Figure 3 illustrates these trends, underscoring the growing disparities in convenience store reliance among counties with older populations or a higher proportion of college-educated residents.

### RQ2: How did COVID-19 impact socio-economic disparities in reliance on fast food outlets?

In terms of summary statistics, reduced fast food reliance of 0.49 (CI: [0.49, 0.49]) was observed during periods of high COVID-19 incidence, compared to 0.57 (CI: [0.57, 0.57]) during periods of low incidence, as detailed in Table 1.

The results from our statistical analysis in Table 5 show that COVID-19 resulted in a significant increase in fast food reliance, with every additional COVID-19 case per 1,000 population increasing the county’s fast food reliance odds relatively by 0.72%.

#### Racial disparities

In terms of summary statistics, Table 1 shows that fast food reliance was lower during periods of high COVID-19 incidence rates in counties across all distinct predominant racial groups. Specifically, in predominantly White counties, fast food reliance was 0.49 (CI: [0.49, 0.49]) during high COVID-19 incidence, which is lower compared to 0.57 (CI: [0.57, 0.57]) during low incidence periods. Similarly, for African American counties, reliance was 0.44 (CI: [0.44, 0.45]) compared to 0.52 (CI: [0.52, 0.53]); in Hispanic counties, it was 0.49 (CI: [0.48, 0.49]) compared to 0.59 (CI: [0.59, 0.6]); in Asian American counties, it was 0.66 (CI: [0.62, 0.71]) compared to 0.86 (CI: [0.82, 0.9]); in Native American counties, it was 0.54 (CI: [0.52, 0.56]) compared to 0.76 (CI: [0.73, 0.8]).

Our statistical analysis, as shown in Table 5, indicates that increases in COVID-19 incidence rates significantly reduced fast food reliance in counties with larger Hispanic populations. Specifically, for every COVID-19 case per 1,000 population, the odds of fast food reliance decreased relatively by 0.003% for each additional percentage point in a county’s Hispanic population. Figure 4 illustrates this trend, showing that counties with higher Hispanic populations exhibit a slower increase in fast food reliance during periods of high COVID-19 incidence compared to periods of low incidence.

#### Other socio-economic disparities

Summary statistics from Table 2 demonstrate lower fast food reliance across counties with distinct predominant household income levels during periods of high COVID-19 incidence. Specifically, in counties where a majority of households earn less than $30,000, fast food reliance was 0.47 (CI: [0.47, 0.47]), lower than 0.54 (CI: [0.54, 0.54]) during periods of low incidence. For counties with household incomes between $30,000 and $60,000, reliance was 0.49 (CI: [0.49, 0.49]), compared to 0.57 (CI: [0.57, 0.58]); for incomes between $60,000 and $100,000, it was 0.5 (CI: [0.5, 0.51]) compared to 0.58 (CI: [0.57, 0.58]); for incomes above $100,000, reliance was 0.52 (CI: [0.52, 0.52]), compared to 0.62 (CI: [0.62, 0.63]).

Stratifying by education levels, Table 3 shows that fast food reliance was higher during high COVID-19 incidence periods across all counties with distinct predominant educational-level groups. Specifically, in counties where the majority of the population holds less than a high school diploma, reliance was 0.62 (CI: [0.6, 0.65]) during periods of high COVID-19 incidence rates, higher compared to 0.51 (CI: [0.46, 0.55]) during low incidence periods. Similarly, for those with a high school diploma, reliance was 0.57 (CI: [0.57, 0.57]) compared to 0.49 (CI: [0.49, 0.49]); for bachelor’s degree, it was 0.78 (CI: [0.76, 0.8]) compared to 0.64 (CI: [0.6, 0.68]); and for advanced degree, reliance was 0.67 (CI: [0.66, 0.68]) compared to 0.58 (CI: [0.56, 0.59]).

Similarly, as shown in Table 4, each population density category showed reductions in fast food reliance during periods of high COVID-19 incidence. For low-density counties, fast food reliance was lower at 0.49 (CI: [0.49, 0.49]) during periods of high COVID-19 incidence, compared to 0.57 (CI: [0.57, 0.57]) during periods of low COVID-19 incidence; for medium-density counties, it was 0.49 (CI: [0.49, 0.49]) compared to 0.57 (CI: [0.57, 0.57]); for high-density counties, it was 0.53 (CI: [0.53, 0.54]) compared to 0.64 (CI: [0.63, 0.64]). Similar trends were observed across age groups, with fast food reliance being lower at 0.48 (CI: [0.48, 0.48]) and 0.5 (CI : [0.5, 0.51]) during periods of high incidence in comparison to 0.56 (CI: [0.56, 0.56]) and 0.58 (CI: [0.58, 0.59]) during low incidence periods for younger counties (under 55) and older (over 55) counties, respectively.

The statistical analysis from Table 5 reveals that heightened COVID-19 incidence rates significantly increased fast food reliance in counties with older populations. Specifically, a 1 per 1,000 population increase in COVID-19 cases raises the odds of fast food reliance relatively by 0.02% for each additional year in the county’s median age. Figure 4 reflects this trend, showing widening disparities in fast food reliance among counties with older median ages.

### RQ3: What socio-economic disparities influence reliance on convenience stores irrespective of COVID-19?

#### Racial disparities

Regardless of COVID-19, Table 5 shows that counties with higher proportions of African American, Hispanic, Native American, and Asian American populations experienced significantly lower convenience store reliance. Specifically, for each additional percentage point in the African American population, the odds of convenience store reliance decreased relatively by 0.52%; in the Hispanic population, by 0.9%; in the Native American population, by 3.7%; and in the Asian American population, by 7.8%.

#### Other socio-economic disparities

Regardless of COVID-19, focusing on the overall trend of convenience store reliance, Table 5 reveals that median age and the proportion of college-educated residents were the only variables to significantly reduce a county’s convenience store reliance. To interpret our results in context: as the median age increases by 1 year, the odds of convenience store reliance decreased relatively by 3.82%, while an additional percent in a county’s college-educated population corresponds to a decrease of 0.56%.

### RQ4: What socio-economic disparities influence reliance on fast food outlets irrespective of COVID-19?

#### Racial disparities

Regardless of COVID-19, Table 5 shows that counties with higher proportions of Hispanics, Native Americans, and Asian American populations experienced significantly higher fast food reliance. Specifically, for each additional percentage point in the Hispanic population, the odds of fast food reliance increases relatively by 0.37%; in the Native American population, by 1.04%; and in the Asian American population, by 1.75%. Conversely, counties with higher proportions of African Americans witnessed significantly lower fast food reliance, with every additional percent in the African American population decreasing the county’s odds of fast food reliance relatively by 0.19%.

#### Other socio-economic disparities

Table 5 shows that irrespective of COVID-19, counties with higher population density, median income, median age, and college education levels have significantly higher fast food reliance. Specifically, for density, every additional 1,000 residents/km^2^ increases the odds of fast food reliance relatively by 1.82%; an increase of every $1,000 in median income corresponds to a 0.7% increase; a 1-year increase in a county’s median age corresponds to a 1.71% increase; and each percentage point increase in college-educated residents corresponds to a 0.60% increase.

## Discussion

### RQ1: How did COVID-19 impact socio-economic disparities in reliance on convenience stores?

The overall decrease in convenience store usage during COVID-19 may be attributed to increased home food preparations, as individuals stayed home more [29]. Consequently, many shopped at grocery stores, with some opting for curbside pickups [11].

Studies have shown that Hispanic communities had reduced mobility, second only to whites [7,8], yet had the most COVID-19 cases relative to other racial groups [8]. This could have heightened their reliance on convenience stores, given their easier access and the increased demand for home essentials.

Counties with a higher concentration of college-educated residents, typically in white-collar jobs, transitioned to working from home during COVID-19. This shift, along with grocery stores’ reduced hours overlapping with regular work hours due to labor shortages [29] and heightened health concerns among the educated [30], probably drove an uptick in convenience store visits.

Similar concerns expressed among the elderly [30], along with their heightened susceptibility to COVID-19 in comparison to youthful populations [31], could potentially explain an increased preference for less crowded and nearer convenience stores among counties with higher median ages.

### RQ2: How did COVID-19 impact socio-economic disparities in reliance on fast food outlets?

The overall increase in reliance on fast food may be associated with the detrimental effects of complete closures faced by sit-in restaurants, particularly during periods of enforced social distancing and lockdowns [32]. As a result, many individuals might have chosen fast food as a handy takeaway option for home consumption.

The heightened dependence on fast food in counties with an older median age can be attributed to COVID-19 attitudes among the elderly, as discussed in the above section. Their concerns about COVID-19 and reluctance to engage in pre-pandemic activities [30] might have encouraged fast food takeaway as a safer option compared to sitting in at full-service restaurants.

Conversely, the reduced reliance on fast food in Hispanic communities might be due to a significant decrease in mobility combined with a disproportionate uptick in COVID-19 cases [7,8], as detailed in the above section. Given that Hispanic counties already had a pronounced dependence on fast foods, as emphasized in Table 1, this decline could be potentially linked to the diminished mobility observed in counties with larger Hispanic populations.

### RQ3: What socio-economic disparities influence reliance on convenience stores irrespective of COVID-19?

Cultural preferences in Asian American and Hispanic communities could potentially explain lower convenience store reliance among these communities. Studies observe a pronounced inclination among Asian Americans for Asian supermarkets [33] and Hispanics for Latino supermarkets [33]. These specialty stores offer imported produce or perishables common in Asian or Hispanic foods [33], which could explain the reduced reliance on convenience stores for dietary needs, especially among communities with a strong Hispanics and Asian-American presence [33].

Similarly, Native Americans in rural tribal communities frequently depend on locally run small-scale grocery stores, even though they are limited in number [34]. While SafeGraph categorizes them as grocery stores due to their size relative to conventional grocery stores, many often lack the fresh and nutritional produce usually available in conventional outlets [34].

Our observation of reduced reliance in counties with higher African American demographics contrasts with some existing studies [1]. Nonetheless, Krukowski et al. [35] note that differences in food store access are more about rurality than racial demographics. This could potentially explain our results, given the urban residence trend among African Americans [36].

Our findings that counties with a higher percentage of college-educated residents relied less on convenience stores were concurred by Lenk et al. [5], potentially due to decreased food desert prevalence [37] and an increased understanding of healthier dietary benefits [38] that are tied to grocery stores [39]. The latter could also explain the decreasing reliance on convenience stores from counties with older median ages [39] as well, in addition to the elderly’s preference for grocery stores as both a shopping and socializing venue [40].

### RQ4: What socio-economic disparities influence reliance on fast foods irrespective of COVID-19?

Overall, counties with larger Hispanic and Native American populations showed increased fast food reliance. This trend is supported by prior research, which indicates a scarcity of full-service restaurants in these communities, creating environments inclined toward fast food consumption [2,3]. Additionally, cultural shifts in younger Asian Americans and Hispanic immigrants [41] underscore these dietary trends. Specifically, the shift from traditional diets to “Americanized” habits might be influencing their food choices more than in older generations [41].

Access to fast food was found to be highest along the U.S. coasts that contain cities with the highest population densities [42]. This might explain the increasing reliance on fast food in more densely populated counties.

Median household incomes also displayed a positive relationship with fast food reliance. This aligns with observations that indicate higher fast food expenditure and lower full-service restaurant consumption among higher-income individuals [43]. However, most studies have suggested the opposite [44].

Similarly, while earlier studies indicated that fast food consumption typically decreased with age [6], a recent study observed a shift in this trend [45]. This correlates with our observation of a positive association between counties with higher median ages and fast food reliance. One potential explanation could be findings that suggest age is negatively correlated with unhealthy perceptions of fast food [46].

Furthermore, our results show an increase in fast food reliance in counties with higher populations of college-educated individuals. This contrasts with some previous findings [6]. However, with recent reports highlighting a massive reliance on fast food among college students [47], these dietary habits formed could have potentially influence long-term dietary behaviors, acting as a catalyst for the observed increase in fast food reliance among college-educated populations.

Conversely, counties with a predominant African American presence showed a decrease in fast food dependence. Previous studies underscore that African Americans frequent fast food and full-service restaurants more than other minority groups [48], as concurred in Table 1. However, studies noted that given a choice, African Americans show a marked preference for casual dining in full-service establishments over fast food [49], which could possibly explain their reduced fast food reliance.

### Policy implications

The rise in fast food and convenience store reliance in counties with older populations is alarming, given seniors’ heightened vulnerability to COVID-19 compared to younger groups [31]. This highlights the importance of ongoing research into the long-term health consequences for the elderly.

Furthermore, Hispanic communities, on top of experiencing the highest number of COVID-19 cases and the second-highest reduction in mobility [7,8], displayed increased reliance on convenience stores. This trend is particularly concerning in light of the deepening obesity disparity among Hispanics [4], particularly given the considerably growing population of counties that already had significant Hispanic populations [50]. These observations highlight the need to strengthen health systems in counties with a predominantly Hispanic population, aiming to combat systemic health inequalities and enhance their preventive health resilience.

### Limitations and future works

Despite the innovative nature of our study, there are limitations. Firstly, while the literature supports our dependent variables by showing a higher prevalence of unhealthy food consumption in fast food and convenience stores [19–21], we lack data on individual food purchases in these locations. This consideration is crucial as unhealthy food consumption ultimately depends on each visitor’s purchase items [11,29]. Additionally, our dataset does not capture food consumption through delivery services, a trend that surged during the pandemic [11,29].

Assessing the overall impact of COVID-19 based solely on its incidence rate does not fully convey its effects on each county for a given month. For instance, restriction measures might have varied across different COVID-19 waves within individual counties. We considered alternative data sources such as Google Mobility Reports, but they covered only 1,718 out of 3,143 counties (56.7%). Using alternative methods, like categorizing by waves or blocks of months, might not capture the diverse levels of restrictions faced in distinct counties. While it is valid to note that responses to COVID-19 might correlate inversely with restriction measures, aggregating incidence rates at a monthly level should suffice in directly reflecting mobility restrictiveness [10,23,24], as detailed in the “Independent variables” section.

Despite these limitations, mobile phone data persist in its ability to reliably and fairly analyze large-scale nationwide data. Therefore, results obtained through mobile phone data are best served when complemented, not replaced, by existing studies, as demonstrated in this study.

### Conclusion

Our study utilized mobile phone data to evaluate COVID-19’s effect on socio-economic disparities related to unhealthy food consumption. We found that the pandemic decreased disparities in fast food consumption in counties with higher Hispanic populations, but exacerbated them in other areas. For example, there was a greater reliance on convenience stores in Hispanic counties and increased reliance on both fast food and convenience stores in counties with older populations. These findings emphasize the importance of fostering resilient and equitable food environments in the post-pandemic era.

## Ethical Approval

Having received only de-identified data and signed an agreement with the data holder, our study was not subject to the Internal Review Board (IRB) federal definitions, confirmed by Washington University’s IRB board.

## Data Availability

SafeGraph provided the mobile phone data used in this study. Restrictions apply to the availability of these data, which were used under license for this study. To access the data from SafeGraph, users can request permission directly from SafeGraph at https://www.deweydata.io/data-partners/safegraph. The COVID-19 data are openly available through the *New York Times* COVID-19 dataset at https://github.com/nytimes/covid-19-data. The racial demographic data are publicly available through the 2017 to 2021 American Community Survey (ACS) census data at https://www.socialexplorer.com/tables/ACS2021_5yr. All codes are publicly available through the following GitHub repository link: https://github.com/cja5553/mobile-phone-data-to-assess-unhealthy-food-reliance

## Supplementary Materials

Supplementary 1 Tables S1 to S4 Detailed explanation of results
