# Supplementary material for: Using Mobile Phone Data to Assess Socio-Economic Disparities in Unhealthy Food Reliance during the COVID-19 Pandemic
Source: Health Data Sci. 2023 Dec 15;3:0101. doi: 10.34133/hds.0101 (PMC10904071; doi:10.34133/hds.0101)
Supplement: Supplementary 1 — Tables S1 to S4 Detailed explanation of results [file hds.0101.f1.pdf]

Supplementary Materials

Supplementary Tables

Table S1: List of other variables that were considered and their respective VIF scores. These variables are omitted due to multi-collinearity, as shown by their high VIF scores.

Consideration of other variables	VIF value range
+ age structure (instead of median age)	1.00 - 8.06
+ all education levels (instead of just % college educated)	1.00 - 8.53
+ occupation composition	1.00 - 9.00

Table S2: Filter criterion for each store type ensuring the reliable classification of convenience stores, grocery stores, fast food restaurants and full-service restaurants.

Store type	Filter criteria	Total chain brands	Total POIs
Convenience stores	Chains classified by SafeGraph as “Convenience Stores” AND Does not contain keywords: (“bar”,“bars”,“deli”,“delis”,“sandwich”, “sandwiches”,“sandwich”,“bakery”,“donut”, “donuts”,“bakerys”, “bakeries”,“cafe”,“cafes”,“coffee”, “coffees”,“pizza”,“pizzas”,“bagel”,“bagels”)	24989	58121
Grocery stores	Classified by SafeGraph as ”Supermarkets and Other Grocery (except Convenience) Stores” AND Does not contain keywords: (”bar”,”bars”,”sandwich”,”sandwiches”,”sandwichs”,”bakery”,”donut”, “donuts”,“bakerys”,“bakeries”,“cafe”,“cafes”,“coffee”,“coffees”,“pizza”,“pizzas”,“bagel”,“bagels”, “furniture”,“furnitures”,“hardware”,“hardwares”,“dicks”,“sport”,“sports”, “electronic”,“electronical”, “electric”,“electronics”,“clothing”,“clothes”,“technology”,“technological”,“technical”,“technology”), WITH THE EXCEPTION of stores containing keywords: (“grocery”,“groceries”,“supermarket”, “supermarkets”,“market”,“markets”,“farm”,“farmers”,“farms”,“produce”)	65227	101676
Fast food restaurants	Chains listed by Technomic as fast food restaurant chains [26]	63	155413
Full-service restaurants	Chains classified by SafeGraph as “full-service restaurants” AND not inside listed within Technomics chains of fast food restaurants [26]	364289	537247

Note: Chain brands are based on `safegraph_brand_ids`, some brands may be classified by SafeGraph under multiple `safegraph_brand_ids`

Table 3: Tests performed to determine the most appropriate model for our statistical analysis.

Dependent variable	Test name	Model comparison (Alternative hypothesis model)	Test statistic
Convenience store reliance	Moran's I test	Panel vs spatial panel model (Spatial panel model)	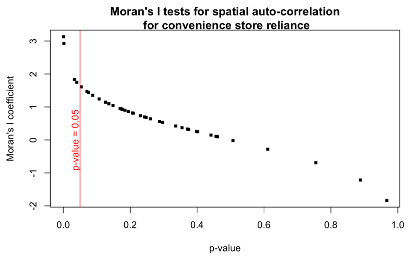
	F-test	Fixed effects vs pooled (Fixed effects model)	61.35***
	Hausman chi-square test	Fixed effects vs random effects (Fixed effects model)	19.46*
Fast food reliance	Moran's I test	Panel vs spatial panel model (spatial panel model)	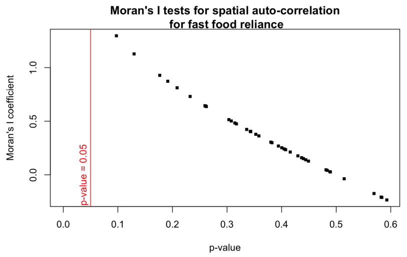
	F-test	Fixed effects vs pooled model (Fixed effects model)	408.15***
	Hausman chi-square test	Fixed effects vs random effects (Fixed effects model)	341.93***

Significant codes: '***' $p < 0.001$, '**' $p < 0.01$, '*' $p < 0.05$

Table S4: Tests performed to check for the statistical assumptions of the model.

Dependent Variable	Test purpose	Test Name	Statistical Value
Convenience store reliance	Multi-collinearity	VIF	1.00 - 2.18
	Normality	Histogram	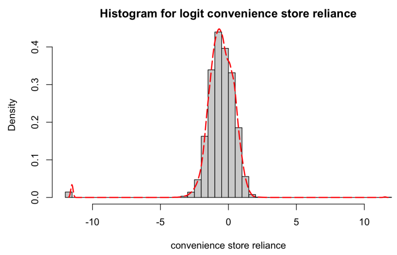
	Homoskedasticity	Breusch-Pagan test	146786 ***
	Cross-sectionality	Breusch-Pagan LM	22853962 ***
		Pesaran CD	268.53 ***
Fast-food reliance	Multi-collinearity	VIF	1.00 - 2.18
	Normality	Histogram	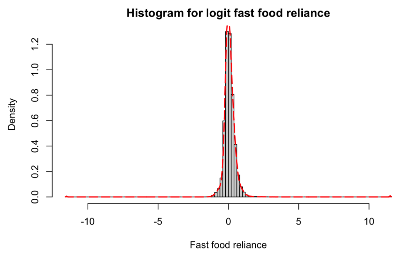
	Homoskedasticity	Breusch-Pagan test	751175 ***
	Cross-sectionality	Breusch-Pagan LM	22853962 ***
		Pesaran CD	268.53 ***

Significant codes: '***' $p < 0.001$, '**' $p < 0.01$, '*' $p < 0.05$

Detailed explanation of results

RQ1: How did COVID-19 impact socio-economic disparities in reliance on convenience stores?

The results from our statistical analysis, presented in Table 5, indicate that COVID-19 resulted in a significant decrease (at $P < 0.001$) in convenience store reliance, with every additional COVID-19 case per 1,000 population decreasing the county's odds on convenience store reliance by 3.41% $(1 - \exp^{-34.719*0.001})$.

Statistical analysis from Table 5 indicates that increases in COVID-19 incidence rates correspond with significant increases in convenience store reliance among counties with higher Hispanic populations. To provide context to our findings: if a county's COVID-19 incidence rate goes up by 1 new case per 1000 individuals, each additional percentage point in the Hispanic population increases the odds of convenience store reliance relatively by 0.01% $(\exp^{13.205*0.01*0.001} - 1)$. This relationship is illustrated in Figure 3, which underscores the widening disparities in convenience store reliance as the Hispanic population percentage grows during periods of high COVID-19 incidence rates.

Increases in COVID-19 incidence rates correspond with significant ($P < 0.001$) increases in counties with higher median ages and higher college-educated demographics. To provide context to our findings: if a county's COVID-19 incidence rate goes up by 1 new case for every 1000 individuals, each additional percentage point in the college-educated population increases the odds of convenience store reliance relatively by 0.02% $(\exp^{23.151*0.01*0.001} - 1)$, whilst an additional year of a county's median age increases the odds of convenience store reliance by relatively 0.06% $(\exp^{0.602*0.001} - 1)$. These changes are illustrated in Figure 3, which reflect widening disparities in convenience store reliance amongst counties of older median ages or higher proportion of college-educated residents.

RQ2: How did COVID-19 impact socio-economic disparities in reliance on fast-food outlets?

The results from our statistical analysis in Table 5 show that COVID-19 resulted in a significant increase (at $P < 0.01$) in fast food reliance, with every additional COVID-19 case per 1,000 population increasing the county's fast food reliance odds relatively by 0.72% $(\exp^{7.191*0.001} - 1)$.

The results from our statistical analysis, as shown in Table 5, indicate that increases in COVID-19 incidence rates correspond with significant ($P < 0.001$) decreases in fast food reliance among counties with higher Hispanic populations. To provide context to our findings: if a county's COVID-19 incidence rate goes up by 1 new case for every 1000 individuals, each additional percentage point in the Hispanic population decreased the odds of fast food reliance relatively by 0.003% $(1 - \exp^{-2.597*0.01*0.001})$. This relationship is illustrated in Fig 4, which shows fast food reliance amongst counties with larger Hispanic compositions increasing at a slower rate during periods of high COVID-19 incidence in comparison to periods of low COVID-19 incidence.

The results from our statistical analysis, as shown in Table 5, show that increases in COVID-19 incidence rates correspond with significant ($P < 0.001$) increases in fast food reliance among counties with higher median ages. Specifically, if a county's COVID-19 incidence rate goes up by 1 new case for every 1000 individuals, each additional year of a county's median age increased the odds of fast

food reliance relatively by 0.02% ($\exp^{0.165*0.001} - 1$). These changes are illustrated in Figure 4, which reflect widening disparities in fast food reliance amongst counties of older median ages.

RQ3: What socio-economic disparities influence reliance on convenience stores irrespective of COVID-19?

Regardless of COVID-19, focusing on the overall trend of convenience store reliance, Table 5 reveals that counties with higher proportions of African Americans, Hispanics, Native Americans, and Asian Americans witnessed significantly lower convenience store reliance (at $P < 0.001$). To interpret our results in context, an additional percentage in the African-American, Hispanic, Native American, and Asian American populations decreased the odds of convenience store reliance relatively by 0.52% ($1-\exp^{-0.523*0.01}$), 0.90% ($1-\exp^{-0.900*0.01}$), 3.7% ($1-\exp^{-3.775*0.01}$), and 7.8% ($1-\exp^{-8.129*0.01}$), respectively.

Additionally, Table 5 reveals that median age and the proportion of college-educated residents were the only variables to significantly reduce a county's convenience store reliance, at $P < 0.001$ and $P < 0.05$, respectively. To interpret our results in context: as median age increases by one year, the odds of convenience store reliance decreased relatively by 3.82% ($1 - \exp^{-0.039}$), whilst an additional percent in a county's college-educated population decreased the odds of convenience store reliance relatively by 0.56% ($1 - \exp^{-0.561*0.01}$).

RQ4: What socio-economic disparities influence reliance on fast foods irrespective of COVID-19?

Regardless of COVID-19, focusing on the overall trend of fast food reliance, Table 5 reveals that counties with higher proportions of Hispanics, Native Americans, and Asian Americans witnessed significantly higher fast food reliance (at $P < 0.01$). To interpret our results in context, an additional percentage in the Hispanic, Native American, and Asian American populations increased the county's odds of fast food store reliance relatively by 0.37% ($\exp^{0.367*0.01} - 1$), 1.04% ($\exp^{1.033*0.01} - 1$), and 1.75% ($\exp^{1.737*0.01} - 1$), respectively. Conversely, counties with higher proportions of African Americans witnessed significantly lower fast food reliance (at $P < 0.001$), with every additional percent in African American population decreasing the county's odds of fast food reliance relatively by 0.19% ($1 - \exp^{-0.188*0.01}$).

Additionally, Table 5 reveals that counties with higher population density, median household income, median age, and proportion of college-educated residents witnessed significant increases in fast food reliance. To interpret our results in context, every additional 1000 residents per square kilometer is expected to increase the odds of a county's fast food reliance relatively by 1.82% ($\exp^{0.018} - 1$), whilst every \$1000 increase in the county's median income is expected to increase the county's odds of fast food reliance relatively by 0.7% ($\exp^{0.007} - 1$). Additionally, every additional year to a county's median age will increase the county's odds of fast food reliance relatively by 1.71% ($\exp^{0.017} - 1$), whilst every additional percent in the county's college-educated demographic will increase their fast food reliance relatively by 0.60% ($\exp^{0.597*0.01} - 1$).
